# Applications of Functional Near-Infrared Spectroscopy (fNIRS) in Studying Cognitive Development: The Case of Mathematics and Language

**DOI:** 10.3389/fpsyg.2018.00277

**Published:** 2018-04-03

**Authors:** Mojtaba Soltanlou, Maria A. Sitnikova, Hans-Christoph Nuerk, Thomas Dresler

**Affiliations:** ^1^Department of Psychology, University of Tübingen, Tübingen, Germany; ^2^LEAD Graduate School & Research Network, University of Tübingen, Tübingen, Germany; ^3^Belgorod National Research University, Belgorod, Russia; ^4^Leibniz-Institut für Wissensmedien, Tübingen, Germany; ^5^Department of Psychiatry and Psychotherapy, University Hospital of Tübingen, Tübingen, Germany

**Keywords:** cognitive development, numerical development, mathematical development, language development, reading acquisition, fNIRS, educational neuroscience

## Abstract

In this review, we aim to highlight the application of functional near-infrared spectroscopy (fNIRS) as a useful neuroimaging technique for the investigation of cognitive development. We focus on brain activation changes during the development of mathematics and language skills in schoolchildren. We discuss how technical limitations of common neuroimaging techniques such as functional magnetic resonance imaging (fMRI) have resulted in our limited understanding of neural changes during development, while fNIRS would be a suitable and child-friendly method to examine cognitive development. Moreover, this technique enables us to go to schools to collect large samples of data from children in ecologically valid settings. Furthermore, we report findings of fNIRS studies in the fields of mathematics and language, followed by a discussion of the outlook of fNIRS in these fields. We suggest fNIRS as an additional technique to track brain activation changes in the field of educational neuroscience.

## Introduction: mathematics and language development

Understanding the processes underlying acquisition and learning of academic skills, such as mathematics and language, are of interest for both educational science and neuroscience. Mastering mathematics and language are central both to career and life perspectives of an individual and also to a society at large (Butterworth et al., 2011). For instance, mathematics has become an inseparable part of everyday life and plays an important role in modern society on every level: from finding a page in a book and selecting a TV channel, to calculating the profits on investments in business and estimating the long-term impacts of political decisions, economic proceedings and social events. Individuals who experience severe difficulties in learning to count and calculate are at a great disadvantage in both academic and professional life (Kadosh and Dowker, 2015). Therefore, the development of numerical abilities is crucial at every stage of the life span from infancy to adulthood (Geary, 2000). Regarding language acquisition, some universal features that are common in all human languages need to be understood by the learning child. This requires a wide-ranging skill set, from domain-specific language-related abilities (e.g., the ability to identify and understand phonemes) to domain-general cognitive abilities (e.g., mental flexibility as bilingual learners switch between languages). Furthermore, languages are built into a social-cultural context; their use is influenced greatly by metalinguistic peculiarities of any society (Obrig et al., 2010), which needs to be taken into account as well.

While multiple valuable behavioral studies of mathematics and language acquisition in childhood have dramatically improved our understanding, in recent years, the educational neuroscience approach has suggested that going beyond behavioral data by means of neurocognitive methods will promote our understanding of cognitive development (Howard-Jones et al., 2016). One neurocognitive method to study cognitive and academic learning and development in children is functional near-infrared spectroscopy (fNIRS). The goal of this review is to outline the contribution of fNIRS to our understanding of the neurocognitive development of mathematics and language skills, particularly in schoolchildren. We briefly explain the concept of educational neuroscience and application of fNIRS. Thereafter, findings of fNIRS studies in these two domains are reported and further discussed.

### Educational neuroscience perspective

In 1997, John T. Bruer wrote a seminal article entitled “Education and the brain: A bridge too far” and concluded back then that we did “not know enough about brain development and neural function to link that understanding directly, in any meaningful, defensible way, to instruction and educational practice” (Bruer, 1997, p. 4). Despite this rather negative conclusion, one needs to be aware that this was about five years after the first functional magnetic resonance imaging (fMRI) and fNIRS studies had been published. Since then neuroscience has progressed rapidly. Over the last decade, neuroscientific methods have been applied to investigate the structural and functional changes in the developing brain across the life span (e.g., Munakata et al., 2004). This has augmented our basic knowledge but is still not directly useful for instruction and educational practice (see below)–despite the initial hope to directly apply neuroscientific findings to learning and teaching strategies. However, the scientific efforts have boosted interest in a new interdisciplinary research field, known as educational neuroscience or neuroeducation. The growth of educational neuroscience has been regarded a two-way street (Geake, 2004), where learning and educational scientists and neuroscientists influence each other (Spelke, 2002; De Smedt et al., 2010).

The field's focus lies on the elucidation of general and specific mechanisms relevant for learning and development. This knowledge, in turn, may help to improve diagnosis and treatment of developmental disorders. It still may have potential for improvements in the current education systems and teaching methods and to make learning most effective according to “sensitive periods” in development (Ansari and Coch, 2006; Goswami, 2006). However, one has to be very realistic and cautious not to predict something unattainable; not everyone agrees that educational neuroscience might contribute to direct innovative educational applications or perspective teaching methods (Bowers, 2016). Recently, there has been a controversial debate over whether neurocognitive data actually aid understanding and facilitation of cognitive development and learning (Bowers, 2016; Gabrieli, 2016; Howard-Jones et al., 2016). Furthermore, few of educators' expectations regarding neuroscience research and how they might find educational neuroscience professionally useful have been met (Hook and Farah, 2013). Thus, the biggest challenges facing educational neuroscience include applying neuroscience findings directly to developmental patterns and educational settings, and improving interdisciplinary communication between teachers and neuroscientists (Ansari et al., 2012).

Different neuroimaging tools have been used to measure underlying neural mechanisms during such cognitive processes as mathematics and language tasks in children. While each tool has specific benefits, its limitations might make it less applicable in developmental populations such as children. As shown in Table 1, fNIRS might be considered one of the most suitable tools to investigate brain activation changes in the frame of educational neuroscience.

**Table 1:** Exemplary advantages and disadvantages of common neurocognitive brain imaging techniques.

**Technique**	**Advantages**	**Disadvantages**
(f)MRI	very high spatial resolution (millimeters) whole brain coverage structural and functional data good source localization	relatively low temporal resolution (seconds) sensitivity to motion artifacts constraints on body position contraindications (e.g., heart pacemaker) expensive
fNIRS	relatively high temporal resolution (milliseconds) recording in natural body positions low sensitivity to motion artifacts portable inexpensive	low spatial resolution (centimeters) only cortical brain coverage influence of extra-cerebral hemodynamics influence of hair and skull characteristics
PET	high spatial resolution (millimeters) whole brain coverage metabolic data	low temporal resolution (seconds) injection of radioactive tracer expensive
EEG	very high temporal resolution (milliseconds) portable inexpensive	low spatial resolution sensitivity to environmental noise inverse problem of source localization time-consuming preparation
MEG	high temporal resolution (milliseconds) high spatial resolution (millimeters) good source localization	sensitivity to environmental noise contraindications (e.g., dental crowns) non-portable expensive

*fMRI, fNIRS, and PET are hemodynamic techniques, EEG is an electrical technique and MEG is an electromagnetic technique. PET, Positron emission tomography; EEG, electroencephalography; MEG, Magnetoencephalography*.

We have scarce knowledge about the neurocognitive foundations of mathematics and language (particularly reading) development in children, in large part due to the limiting factors of commonly used neuroimaging tools. Given that one of the main aims of educational neuroscience is to advance diagnostic and interventional approaches in learning disabilities, a suitable tool should allow us to measure these cognitive processes in natural settings, such as in schools (Mücke et al., 2018).

### FNIRS as an imaging technique

**Figure 1:**
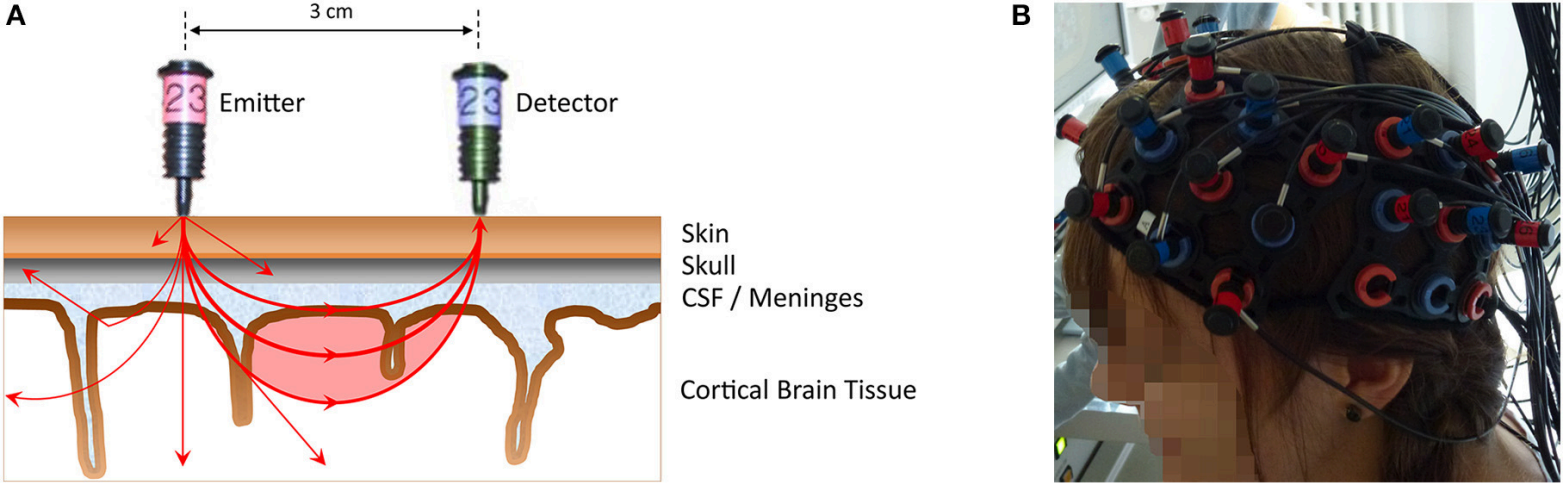
**(A)** A simplified illustration of an emitter-detector optode pair (i.e., one measurement channel) representing the principles of NIRS measurement (distance 3 cm, Hitachi ETG-4000). Near-infrared light from the emitter (red optode) penetrates the scalp to pass through different biological tissues (e.g., skin, skull, CSF [cerebro-spinal fluid]/meninges, cortical brain tissue). The near-infrared light that is subsequently detected (blue optode) on average travels through a “banana-shaped” form (red-shaded area), allowing hemodynamic changes within this area to be assessed. Note that due to the properties of the penetrated medium (e.g., resulting in scattering, reflection, absorption by oxygenated, and deoxygenated hemoglobin), only a fraction of the emitted light reaches the detector. This is illustrated by exemplary photon paths on the left side. From the intensity loss at the detector site, concentration changes in oxygenated and deoxygenated hemoglobin can be calculated. The near-infrared light originating from one emitter can be detected by several detectors surrounding that emitter, thus resulting in neighboring channels (e.g., photons propagating to the left). **(B)** The placement of a multi-channel fNIRS probe sets.

FNIRS is an optical imaging technique that uses near-infrared light (wavelengths 650–950 nm) to measure concentration changes of oxygenated and deoxygenated hemoglobin in cortical brain structures (for more in-depth reviews see Ferrari and Quaresima, 2012; Scholkmann et al., 2014). Light from the near-infrared range has the ability to penetrate biological tissue (e.g., skin, skull, brain) and is mainly absorbed by oxygenated and deoxygenated hemoglobin. With the most commonly used continuous-wave systems, near-infrared light is continuously sent via light emitters (emitting optodes) through brain tissue and then collected by light detectors (detecting optodes). A pair of emitter-detector optodes represents a measuring channel. A simplified illustration can be found in Figure 1A. The average trajectory of photons from the emitter to the detector can be represented by a “banana-shaped” form that in part pervades cortical tissue (red-shaded area in the figure). Owing to the above-mentioned absorption and scattering of near-infrared light, there is a loss of light intensity at the detector. Using the modified Beer-Lambert Law for light attenuation in scattering biological tissue and specific assumptions, concentration changes of oxygenated and deoxygenated hemoglobin can be calculated from the intensity loss. Technically, fNIRS does not assess brain activity directly, but measures concentration changes of oxygenated and deoxygenated hemoglobin in the blood vessels. As neuronal activity leads to an increase in regional blood flow, combined with an increase in oxygenated and decrease in deoxygenated hemoglobin (neurovascular coupling), neural activity can be inferred from the concentration changes of the chromophores. Hence, similar to fMRI, fNIRS measures an optical blood oxygen level dependent (BOLD) signal, which can be analyzed using methods similar to those applied to fMRI data (i.e., general linear model, channel-wise analysis, region-of-interest analysis, and functional connectivity). These days, with the availability of multi-channel systems (cf. Figure 1B), fNIRS can be considered cost-effective, in comparison with fMRI, as a non-invasive brain imaging technique that offers several additional advantages (cf. Table 1). These advantages make it suitable for investigating neurocognitive measures in ecologically valid settings such as schools or kindergartens with a natural response type—a verbal or written production paradigm—which is the core concept of the current review. Similar to the other neuroimaging techniques, it also facilitates the study of questions of innateness, as neural responses to language or numerical stimuli can be measured in the absence of conscious language understanding or verbal or manual responses.

Several commercial NIRS machines are available, which differ regarding various parameters such as time resolution, number of emitters/detectors, adjustable, or fixed emitter-detector-distances, employed wavelengths, etc. (see Scholkmann et al., 2014).

FNIRS and fMRI (the most common neuroimaging technique) should be considered complementary techniques, since both have their advantages and disadvantages. The use of a specific method should always be dependent on the research question and the respective samples. In a sample without anxiety and specific MRI contraindications, or in simple research paradigms without movement artifacts, fMRI may be the better choice. In young children and especially in tasks where movements are present, fNIRS may be the better choice. In this article, we concentrate on the contribution of fNIRS to two major fields of cognitive and educational development: mathematics and language.

## Application of fNIRS to the investigation of mathematics

FMRI studies showed that in infants and preschoolers the right parietal cortex is sensitive to changes in the cardinality of a set of objects (Cantlon et al., 2006; Izard et al., 2009; Park et al., 2014). Activation shifts progressively from the right intraparietal sulcus to bilateral intraparietal sulcus in numerical-magnitude processing due to the acquisition of the exact number system (Ansari et al., 2006; Cantlon et al., 2006; Piazza et al., 2007; Emerson and Cantlon, 2015). Moreover, there is a developmental fronto-parietal shift representing the change from more effortful procedural strategies to more automatic and retrieval strategies between 8 and 19 years of age (e.g., Rivera et al., 2005). This shift accompanies a decreased activation of the hippocampus in adults and adolescents compared to 8- to 9-year-old children (Qin et al., 2014) and an increased activation of the left supramarginal gyrus and angular gyrus by involving language-related areas in retrieving facts from long-term memory between 8 and 14 years of age (Ansari, 2008; Prado et al., 2014). However, because of scarce neuroimaging studies in children, it is not easy to distinguish which changes are due to specific mathematical learning and general cognitive development, and which are due to the maturation of the brain (Arsalidou et al., 2017; Peters and De Smedt, 2017). Moreover, the generalization of such findings in experimental settings to ecological settings is not trivial since mathematics in real life is not conducted lying down in a noisy environment without movement, which can affect numerical learning.

FNIRS research in this field has only started to emerge in recent years as it allows overcoming some of the aforementioned challenges. For example, fNIRS studies revealed activity in the right intraparietal sulcus—a key region for number processing—in response to a numerical change in a visual pattern in awake 5.5–6.5-month-old infants (Hyde et al., 2010; Edwards et al., 2016). In other words, this technique makes it possible to see the brain regions responding in awake infants. In the study by Hyde et al. (2010), infants were adapted to a set of 16 dots and in an oddball paradigm they were shown images changing in numerosity (8 or 16 dots) and shape (16 squares or triangles). In the study by Edwards et al. (2016), they were shown either the same number of dots (8 or 16) or sets varying in numerosity (8 and 16) in different blocks. In both studies, an increased activation was found in the right intraparietal sulcus in response to the change in the number of dots, in comparison with other conditions. These findings provided evidence that infants start mentally operating with non-symbolic numerosity very early, and rely on their approximate number system before acquiring language and symbolic number system experience. As for neural correlates of numerical cognition in preschoolers, there are no fNIRS studies yet.

FNIRS studies of arithmetic performance in primary- and secondary-school children (e.g., Dresler et al., 2009; Soltanlou et al., 2017a) revealed bilateral activation of a frontal-parietal network, the network that has been observed in fMRI neuroimaging studies in adults and children (Arsalidou and Taylor, 2011; Arsalidou et al., 2017). Dresler et al. (2009) presented an arithmetic problem either in numeric format or embedded in text to primary- and secondary-school children (cf. Table 2). In this study, a sample of 90 children was measured, which is very difficult to achieve with other techniques such as fMRI. They observed greater activation in parietal and posterior frontal regions for the calculation than for reading in both primary- and secondary-school children, which is in line with fMRI results in children (Rivera et al., 2005; Ansari et al., 2006; Kucian et al., 2006) and fNIRS results in adults (Richter et al., 2009; but see Artemenko et al., 2018a for basic tasks of copying numbers and letters). Moreover, greater task-related activation in bilateral frontal areas (precentral premotor and motor areas) was observed in younger children than in older children. This activation is due to less automatized calculation processing and more speech-related activity. In line with previous studies (Rivera et al., 2005; Kaufmann et al., 2006; Kucian et al., 2008), this fNIRS finding in schoolchildren pointed out a developmental frontal-to-parietal shift in brain activation.

**Table 2:** Summary of fNIRS studies investigating mathematics in schoolchildren.

	**Study**	**N**	**M_age_**	**Age range**	**Task**	**Conditions/Factors**	**Main findings**
1	Çiftçi et al., 2008	14	n/a	15–16	subtraction	task (calculation vs. rest)	right frontal activation increased during calculation compared with rest
2	Kuroda et al., 2009	8	11.88	11–12	geometry	solution strategies (no strategy, developed strategy, applied strategy)	prefrontal activation increased in children who could not develop a problem solving strategy; prefrontal activation reduced in children who developed a strategy; no activation changes in prefrontal cortex were observed in children who had already developed a strategy before solving the problems
3	Dresler et al., 2009	46 44	9.98 13.88	9–11 13–15	addition	format (numeric vs. word problem) task (calculation vs. reading) hemisphere (left vs. right) grade (4th vs. 8th)	calculation resulted in greater activation in parietal and posterior frontal regions compared to reading; neither format nor age had a significant effect on brain activation
4	Obersteiner et al., 2010	46 44	9.98 13.88	9–11 13–15	addition	format (numeric vs. word problem) grade (4th vs. 8th) competency (low, average, high) complexity (carry vs. no-carry)	parietal activation during calculation was not affected by age, mathematical competency, or task characteristics; marginally greater activation of parietal areas was reported for word problems as compared to numeric problems
5	Soltanlou et al., 2017a	24	11.10	10–13	multiplication	complexity (one-digit vs. two-digit)	activation increased in the left superior parietal lobule, intraparietal sulcus, and postcentral gyrus for one-digit multiplication problems; activation increased in bilateral superior parietal lobule, intraparietal sulcus, middle frontal gyrus, left inferior parietal lobule for two-digit multiplication; greater activation was reported in the right middle / inferior frontal gyrus for two-digit multiplication as compared to one-digit multiplication
6	Soltanlou et al., 2018	20	11.10	10–13	multiplication	training duration (one-session and 2-week) training (trained vs. untrained) complexity (one-digit vs. two-digit)	one-session training led to a decreased activation at the left angular gyrus and right superior parietal lobule and right intraparietal sulcus for complex multiplication; 2-week training led to a decreased activation at the left angular gyrus and right middle frontal gyrus in complex multiplication; reduced activation of the fronto-parietal network was reported; there is no support of the left angular gyrus in arithmetic learning in children
7	Artemenko et al., 2018b	19 19	12.19 13.34	11–1312–14	4 basic arithmetic operations	operation (addition, subtraction, multiplication, division) grade (6th vs. 7th) complexity (one-digit vs. two-digit)	a common bilateral fronto-parietal network was activated for all basic arithmetic operations, similar to adults; during one year, frontal activation decreased for subtraction while angular gyrus and temporal activation increased for addition and multiplication; an inverse effect of arithmetic complexity was reported for parietal activation in multiplication and for the left angular gyrus and temporal activation in addition

*Study 1 and 2 were published in a proceedings journal. Study 4 is a reanalysis of data of study 3. Study 5 and 6 were combined fNIRS-EEG studies in the same children. Study 7 is longitudinal*.

This developmental shift was also reported in another longitudinal fNIRS study (Artemenko et al., 2018b). The applicability of fNIRS in a natural setting let them measure brain activation during all four basic operations in a written production paradigm. Because of uncomfortable positioning in the fMRI scanner and sensitivity of this technique to movement artifacts, most fMRI studies have not used written production, which is the most ecologically valid way to solve such tasks, as they would be solved in schools. Artemenko et al. (2018b) reported decreased activation of frontal regions for subtraction—less effortful calculation—from 6th to 7th grade and at the same time increased activation of the angular gyrus and temporal regions for addition and multiplication—more automatic calculation and fact retrieval (cf. Table 2). Importantly, in such a natural setting, they not only found a shift between activation regions of interest, but also more efficient (or less effortful) processing within those regions.

However, in children, not only the operation and the age play a role in neurocognitive processing in arithmetic, but also the mathematical competency. Obersteiner et al. (2010) investigated the fNIRS data of Dresler et al. (2009) in more detail and besides the format and grade further explored mathematical competency (low, average, high) and task complexity (addition with and without a carry operation) in the calculation condition (cf. Table 2). For these factors, no significant activation differences were found in the targeted parietal regions, with a trend for higher activation for word than for numeric problems. However, they observed higher parietal activation in no-carry addition than carry addition in the group with average mathematical competency. Interestingly, this study was conducted at school, which is impossible in the case of several non-portable neuroimaging techniques such as fMRI. This situation probably leads to less anxiety in children then when they come to experimental labs, which might influence the activation patterns.

In another fNIRS study in secondary-school children, Kuroda et al. (2009) utilized fNIRS to measure brain activation changes during spatial manipulation with objects, which is important for geometry (cf. Table 2). Prefrontal activation was measured in 6th graders while they were solving tangram puzzle tasks. Different activation patterns were detected in accordance with strategies the children used to solve problems, which led to three groups of children based on their solution methods. In children who were not able to solve the tangram puzzle, prefrontal activation continually increased. In children who were able to develop a strategy in the process of manipulating tangram pieces, prefrontal activation steadily declined. No changes in prefrontal activation were found in a group where children had already developed a strategy before solving the problems. In general, the findings are in line with neuroimaging studies of prefrontal activation: when the level of task complexity increases, the activation may increase correspondingly (Kuroda et al., 2009; see also Mücke et al., 2018). Again, the possibility of testing in a natural setting let them measure brain activities during tangram puzzle solving (see also Soltanlou et al., 2017b), which cannot be done in an fMRI scanner.

In a recent combined fNIRS-EEG study, Soltanlou et al. (2017a) investigated arithmetic complexity in multiplication problems in 5th graders. This study shows the feasibility of fNIRS in combination with other techniques such as EEG, which does not lead to additional artifacts in EEG signals (cf. Table 2). Soltanlou et al. (2017a) observed significant activation in the left superior parietal lobule, intraparietal sulcus, and postcentral gyrus for one-digit multiplication problems, while bilateral superior parietal lobule, intraparietal sulcus, middle frontal gyrus, and left inferior parietal lobule were activated when children were solving complex multiplication problems. The contrast of complex vs. simple revealed greater activation in the right middle frontal gyrus but not in parietal regions. This finding shows that in children, increasing mathematics complexity promotes domain-general cognitive processes, i.e., working memory, sustained attention and planning (see also Mücke et al., 2018). Furthermore, the authors suggested that at this stage in development, children rely on domain-specific magnitude processing for both simple and complex calculations (Soltanlou et al., 2017a). This finding was in line with the findings of Artemenko et al. (2018b) showing a decrease in activation of middle and inferior frontal gyri from 6th to 7th grade. Therefore, we might conclude that dependency on domain-general cognitive processes in mental calculation declines during development. However, another fNIRS study of mental calculation in high-school children between the ages of 15–16 years (Çiftçi et al., 2008) revealed greater activation of the right prefrontal cortex during subtraction compared to rest (cf. Table 2). This finding points out that improvement in solving subtraction problems relies on fast procedural processes rather than fact retrieval processes (Prado et al., 2014) because adolescents still rely on this activation to solve subtraction problems but are fast in problem solving.

In another fNIRS-EEG learning study on multiplication problems with 5th graders, Soltanlou et al. (2018) reported decreased activation of the right middle frontal gyrus during trained vs. untrained sets after 2 weeks of training (cf. Table 2), which was in line with previous arithmetic learning studies in adults (Zamarian et al., 2009) and children (Arsalidou et al., 2017; Peters and De Smedt, 2017). Additionally, the authors found decreased activation of the left angular gyrus in trained vs. untrained sets via multiplication learning in children, which was contradictory to adults' studies (Soltanlou et al., 2018). This finding is line with a recent meta-analysis showing that a brain activation network underlying arithmetic processing and development in children differs from that in adults (Arsalidou et al., 2017). Moreover, they argue that one of the aims of this study was to investigate brain activation changes due to training in an ecologically valid setting.

To conclude, the consistency of the findings in the few above-mentioned fNIRS studies with the findings of other approaches, particularly fMRI, in schoolchildren (Peters and De Smedt, 2017), reveals the feasibility of this approach in educational neuroscience. Usually, a frontal-parietal network is observed, which varies with task complexity, age, and the expertise of the children. Greater task complexity, younger age, and lesser expertise usually require more effortful processing and more involvement of frontal regions, corresponding with domain-general contributions to arithmetic problem solving for such groups and problems.

## Application of fNIRS to the investigation of language

FMRI studies showed that the major brain regions responsible for many aspects of language development and processing make up the left perisylvian area in human cortex, including Broca's and Wernicke's areas (Gazzaniga, 2004). The brain specialization for language acquisition in neonates and infants is subserved by a temporo-frontal loop. Thereafter, when reading skills are acquired in children aged from 7 to 17 years, visual forms of words are represented in the occipital-temporal areas (Shaywitz et al., 2002). Interestingly, the processing of written and spoken words similarly rely on posterior multi-modal areas including Wernicke's area (Booth et al., 2001). However, understanding and appropriately using language in different contexts, evaluating humor and emotional expressiveness, together with visuospatial processing, which is needed for reading skill, also involve the right hemisphere or other brain areas in the left hemisphere (Kensinger and Choi, 2009). In sum, while a left lateralization for language can be already observed in infancy, the right hemisphere also plays an essential role in some aspects of language processing and reading in particular. However, because of the advantages of fNIRS, it is worthwhile to measure these processes in more natural settings to test the feasibility of this generalization.

During the last decade, fNIRS has been used in several language studies conducted with neonates, infants, children and adults (for a review see Minagawa-Kawai et al., 2008; Ferrari and Quaresima, 2012; Quaresima et al., 2012; Vanderwert and Nelson, 2014; Aslin et al., 2015). FNIRS has been successfully applied to study neural correlates of linguistic and non-linguistic processing in native and non-native languages in newborns (Pena et al., 2003; Telkemeyer et al., 2009; Arimitsu et al., 2011; May et al., 2011; Vannasing et al., 2016) and 3- to 11-month-old infants (Homae et al., 2006, 2007). The ability to perceive acoustic and speech stimuli helps infants process segmental and suprasegmental information from birth. It was shown that the auditory cortex of neonates is sensitive both to phonemic and prosodic information, but with different patterns of brain activation. Arimitsu et al. (2011) revealed a right-dominant superior temporal sulcus and mid temporal gyrus activation in neonates (3–8 days old) in response to intonation changes in speech (eg., itta vs. itta?), and a left-dominant activation in temporal and inferior parietal regions (supramarginal gyrus) in response to the phonemic changes (e.g., itta vs. itte) due to the verbal-auditory short-term memory. FNIRS has been also successfully combined with other neuroimaging and neurophysiological techniques to study language. For instance, in a combined fNIRS-EEG study in 2- to 6-day-old neonates, Telkemeyer et al. (2009) found an increased activation in right inferior and posterior temporal areas in response to prosodic information, and a left hemisphere (temporoparietal) dominance for fast acoustic modulations that are most relevant to phonetic processing. In sum, the results converge to show that newborns process language properties bilaterally, and activation is mostly observed in temporal and inferior parietal regions known as language areas from adult studies. Studies using fNIRS with infants and preschoolers revealed similar results (Homae et al., 2006, 2007, 2011; Wartenburger et al., 2007; Telkemeyer et al., 2011).

In a combined fNIRS-EEG study of 3- and 6-month-old infants, Telkemeyer et al. (2011) observed right temporal activation in response to different changes in prosodic information (slow modulations), while left tempo-parietal regions responded to speech processing (fast modulations). Later, at the age of 4 years, this right lateralized pattern of processing the prosodic components of speech remained significant (Wartenburger et al., 2007). Wartenburger et al. (2007) showed that linguistic information is processed in left fronto-temporal regions, whereas prosodic information engages right fronto-temporal activation. Similar findings (cf. Table 3) were observed in older children at the ages of 6–9 years (Kovelman et al., 2012). These fNIRS findings were interpreted as showing a tendency of the right hemisphere to process slow rhythmic stimuli, and a selective sensitivity of the left hemisphere to a specific range of slow rhythmic modulations that are important for reading acquisition (Kovelman et al., 2012). These slow modulations correlate with reading ability in 10-year-old children (Goswami, 2011). These findings suggest that the prosodic processing is innate or at least very quickly developed, and the ability to use it to identify utterance enhances during the first year. However, this processing changes with age: the more linguistic information is processed, the more the left hemisphere is involved. Moreover, these findings demonstrate that suprasegmental information plays a crucial role even at an early age and corresponds to adult-like activation in response to intonation or loudness of the speaker (Meyer et al., 2004; Obrig et al., 2010). Therefore, for language as for numerosity development, fNIRS is especially suited to investigate innate or quickly emerging neural responses to language in the absence of any language production in infants.

**Table 3:** Summary of fNIRS studies investigating language in schoolchildren.

	**Study**	**N**	**M_age_**	**Age range**	**Task**	**Conditions/Factors**	**Main findings**
1	Sugiura et al., 2011	484	8.93	6–10	word repetition	language (Japanese vs. English) word frequency (high vs. low) hemisphere (left vs. right)	native language elicited greater activation in superior / middle temporal and angular / supramarginal gyri; dominant activation of the right inferior frontal gyrus; low-frequency words elicited more activation in the right supramarginal gyrus, while high-frequency words elicited more activation in the left angular gyrus; involvement of the right hemisphere while acquiring unfamiliar/low-frequency words; a right-to-left shift in inferior parietal lobule as lexical knowledge increases irrespective of language
2	Kawakubo et al., 2011	48 22	10.90 27.30	5–18 21–37	letter fluency	age (children/adolescents vs. adult) gender (male vs. female) hemisphere (left vs. right) letter frequency as covariate	developmental change with age in the frontopolar regions (BA9/10); larger oxygenated hemoglobin changes in adult males than child/adolescent males; gender difference in activation of the frontopolar prefrontal cortex in adulthood but not from childhood to adolescence
3	Tamekuchi et al., 2011	8 10	7.40 39.20	6–9 35–44	verbal fluency	task vs. baseline	greater activation in the left prefrontal cortex in children; greater activation in the right prefrontal cortex in adults
4	Kovelman et al., 2012	15	7.00	6–9	rhythm perception phonological awareness rhyming word matching	task vs. rest frequency (0.5, 1.5, 3.0 Hz) hemisphere (left vs. right)	greater activation of right hemisphere toward the slow rhythmic stimuli, and left hemisphere toward both faster and slower frequencies; overall better ability to process rhythmic sensitivity in right hemisphere, while a select sensitivity to a preferred range of slow rhythmic modulations in left hemisphere, which might be responsible for cross-modal language processing and reading acquisition
5	Jasinska and Petitto, 2013	20 bc 20 mc 10 ba 9 ma	8.90 8.92 19.90 19.00	7–10 7–10 17–26 17–24	sentence judgment	age (children vs. adults) language (monolingual vs. bilingual) bilingualism (early-exposed vs. later-exposed) hemisphere (left vs. right) sentence type (object-subject vs. subject-object)	greater activation in the language areas, superior temporal gyrus, inferior frontal gyrus in right hemisphere in bilingual children and adults; greater activation in classic language areas in early-exposed, while greater activation of prefrontal cortex in later-exposed; modification of the language processing areas via early-life language experiences
6	Jasinska and Petitto, 2014	8 mc 8 mc 8 ma 8 bc 8 bc 8 ba	7.70 9.30 18.90 7.60 9.20 18.60	6–8.5 8.5–10 18–20 6–8.5 8.5–10 18–20	single-word reading	age (younger children, older children, adults) language (monolingual vs. bilingual) word regularity (regular, irregular, non-sense)	age-related shift in the left inferior frontal gyrus and superior temporal gyrus; greater and more variable activation in bilateral inferior frontal gyrus and superior temporal gyrus, dorsolateral prefrontal cortex, and rostrolateral prefrontal cortex; modification of the neural systems underlying reading development through different early-life language experience
7	Tando et al., 2014	9 9 10 9 9	7.60 10.70 13.50 17.50 34.80	6–8 9–11 12–14 15–18 n/a	verbal fluency	task vs. rest age (5 groups) time course (4 times)	increasing activation of frontopolar region in prefrontal cortex with age; decreased performance with time in all groups; beginning of maturation of verbal retrieval functions in early adolescence
8	Sugiura et al., 2015	484	8.93	6–10	word repetition	language (Japanese vs. English) word frequency (high vs. low) gender (male vs. female) hemisphere (left vs. right)	native language elicited greater activation in superior / middle temporal and angular / supramarginal gyri; activation of the angular and supramarginal gyri during high-frequency word in males but not in females
9	Paquette et al., 2015	10 10 11 9	5.00 8.60 13.82 24.00	3–6 7–10 11–16 19–30	verbal fluency	task vs. rest age (4 groups) hemisphere (left vs. right) brain region (Broca vs. Wernicke)	greater activation in left hemisphere, with weaker activation in right hemisphere during task; increased activation in bilateral hemisphere during the task with age; left hemisphere specialization for language from early childhood
10	Tellis and Tellis, 2016	50	21.90	11–52	silent and aloud readingfree speech finger tapping	gender (male vs. female) task (silent reading, reading aloud, free speech, finger tapping) hemisphere (left vs. right) brain region (BA 44&45, BA 21,22,39,40,41&42)	greater activation in bilateral frontal regions during free speech; no gender differences; significant differences in right superior temporal gyrus and primary auditory association cortex between tasks; significant differences in left supramarginal gyrus between tasks
11	Gallagher et al., 2016	6 8 11 8	4.67 8.75 14.27 24.75	3–6 7–10 11–16 20–30	verbal fluency	task vs. rest age (4 groups) hemisphere (left vs. right) brain region (bilateral frontal and temporal areas)	greater activation in left hemisphere for language networks both during the task and at rest; a very good concordance between functional connectivity and conventional results was observed
12	Jasinska et al., 2017	11 mc 7 bc1 6 bc2	8.09 8.00 8.25	7–9 7–9 7–9	word reading	language (monolingual, bilingual 1, bilingual 2) words (irregular, regular, pseudo-words) brain region (left inferior frontal gyrus vs. left superior temporal gyrus)	greater activation in left posterior temporal regions associated with direct sound-to-print phonological analyses in bilinguals; greater activation in left frontal regions associated with assembled phonology analyses in bilinguals; significant impact of bilinguals' two languages on children's neurocognitive architecture for learning to read
13	Walsh et al., 2017	16 sc 16 cc	9.10 9.20	7–11 7–11	picture description task	group (stuttering children vs. controls) hemisphere (left vs. right) brain region (inferior frontal gyrus, premotor cortex, superior temporal gyrus)	deactivation in left dorsal inferior frontal gyrus and premotor cortex in stuttering children as compared to control children
14	Mücke et al., 2018	50	10.60	10–11	semantic and phonetic verbal fluency mental arithmetic task	group (high vs. low moderate-to-vigorous physical activity) task (semantic verbal fluency, phonetic verbal fluency, mental arithmetic)	no group differences in response to the cognitive tasks; greater bilateral increase in the anterior prefrontal region during the semantic verbal fluency; increase in the left anterior prefrontal region in response to the phonetic verbal fluency
15	Groba et al., 2018	18 bc3 28 mc	4.98 5.00	4–6 4–6	adjective learning task with descriptive hand gesture	language (monolingual vs. bilingual) hemisphere (left vs. right) brain region (prefrontal, frontal, fronto-temporal, temporal and temporo-parietal)	greater activation in right superior temporal sulcus in bilinguals during learning of adjectives due to heightened pragmatic sensitivity

*Bc, bilingual children; mc, monolingual children; ba, bilingual adults; ma, monolingual adults; sc, stuttering children; cc, control children; bc1, Spanish-English; bc2, French-English; bc3, Spanish-German. Study 8 is a reanalysis of data of study 1*.

Some characteristics of fNIRS, such as robustness to muscle movements in the case of reading and speaking (speech production) can be considered a significant advantage in neuroimaging studies of language development in preschoolers and schoolchildren (Gallagher et al., 2016; Walsh et al., 2017). For instance, Tellis and Tellis (2016) measured brain activation changes during three different reading tasks in children and adults: silent reading, reading out loud, and free speech on a given topic. They observed the highest activation during free speech in bilateral frontal regions. In another example, Kawakubo et al. (2011) observed developmental changes from 5 to 37 years in the frontopolar region during a letter fluency task (see also Tamekuchi et al., 2011). Furthermore, they found a gender difference in the frontopolar region in adulthood, which was not observed in childhood or adolescence (Kawakubo et al., 2011). Similar results were found in an fNIRS study by Tando et al. (2014), who observed increased activation of the frontopolar region during a verbal fluency task from 6 to 18 years (cf. Table 3). They concluded that maturation of the verbal retrieval functions starts in early adolescence and continues into adulthood (Tando et al., 2014). Moreover, Tamekuchi et al. (2011) reported higher activation in the left prefrontal cortex in children (6–9 years) than in adults (35–44 years) during a verbal fluency task, while the opposite pattern was observed in the right prefrontal cortex. In line with this finding, Paquette et al. (2015) investigated age-related changes in lateralization of brain activation during expressive language from 3 to 30 years. However, they observed a developmental increase in both hemispheres during a verbal fluency task (Paquette et al., 2015). They found greater activation in left temporal and frontal areas compared with right hemispheric areas in all ages, and concluded that left hemispheric specialization for expressive language is established in very young children and develops until adulthood (Paquette et al., 2015). Interestingly, Walsh et al. (2017) observed deactivation in the left hemisphere, i.e., dorsal inferior frontal gyrus and premotor cortex during speech production in 7- to 11-year-old children with stuttering compared to typically developing peers. The above-mentioned tasks with verbal production are rarely used in fMRI studies, while they can be readily utilized during fNIRS measurement (cf. Table 3).

The application of fNIRS to the detection of brain mechanisms of bilingual children has been another interesting area for language studies. Jasinska and Petitto (2014) investigated the neural basis for reading by applying tasks with three word-type conditions (regular, irregular, and nonsense words). They tested two groups of monolingual and bilingual primary-school children: younger (ages 6–8) and older (ages 8–10) in comparison to adults (cf. Table 3). Younger children showed bilateral superior temporal gyrus activation for both regular and irregular words due to a high level of control of matching phonological processing and orthography. Older children revealed left inferior frontal gyrus activation for irregular in comparison to regular words, and in contrast, inferior parietal lobule activation for regular in comparison to irregular words, due to paying attention to lexical word complexity and whole-word processing in general (Jasinska and Petitto, 2014). Compared to monolingual readers, bilinguals in all age groups showed greater bilateral activation in classic language areas (left inferior frontal gyrus, superior temporal gyrus, and inferior parietal lobule), and homologous areas in the right hemisphere. They also revealed greater activation in the prefrontal cortex including the rostrolateral prefrontal cortex and dorsolateral prefrontal cortex due to employing such cognitive processes as reasoning, working memory, and attention, which are necessary for switching between languages. This finding is further supported by work showing increased activation of bilateral prefrontal cortex during a verbal fluency test in 10- to 11-year-olds (Mücke et al., 2018). Bilingual children are supposed to have greater cognitive plasticity, better sensitivity to language functional and structural peculiarities, and more flexibility in their way of thinking than many monolingual children between the ages of 5 and 10 years (Bialystok et al., 2012; see also Groba et al., 2018). For instance, Jasinska et al. (2017) observed greater activation in left posterior temporal regions—associated with direct sound-to-print (transparent) phonology—and decreased activation in left frontal regions—associated with assembled phonology—in Spanish-English and French-English bilingual children from ages 6 to 10, but not in English monolingual children during an overt word reading task (cf. Table 3). The fNIRS findings suggest that these neural circuitries are highly relevant for reading skills in bilinguals (Jasinska et al., 2017). In line with these findings, Jasinska and Petitto (2013) observed greater activation in left-hemispheric language areas, and additionally, right-hemispheric homologs, i.e., right superior temporal gyrus, and inferior frontal gyrus, in bilingual children (ages 7-10) and adults as compared with monolinguals (cf. Table 3).

In a large-sample study, 484 elementary-school children (6- to 10-year-olds) performed word repetition tasks in their native language and second language, while their brain activation was recorded by means of fNIRS in a neuroimaging vehicle (Sugiura et al., 2011), showing the portability of fNIRS devices. FNIRS findings revealed that the cortical activation pattern associated with language processing involved a bilateral frontal, temporal, and parietal network (Sugiura et al., 2011). Native language words evoked significantly greater left brain activation than foreign words in the superior and middle temporal gyri, and in inferior parietal regions (angular and supramarginal gyri). Foreign language words elicited activation in the right hemisphere, as primary-school children had only started learning foreign languages and did not know many non-native words (see also Groba et al., 2018). Moreover, low-frequency words in both languages led to significant activation in the right supramarginal gyrus, while left-sided activation was detected in the angular gyrus for high-frequency words in the native language, as the lexical meanings of most these words were familiar to children. Sugiura et al. (2015) showed that high frequency word processing leads to increased activation in the angular and supramarginal gyri in boys but not in girls. While the main effect of sex in these two regions was significant, its interaction with hemisphere did not reach significance level. They suggested that this activation differs between boys and girls (Sugiura et al., 2015). This tendency shows the right-to-left shift in the inferior parietal areas when lexical proficiency increases in acquiring knowledge in any language. Sugiura et al. (2011) also found a statistically significant tendency in the age-related dynamics: the older the children were, the less activation was detected in auditory and temporal areas for the native language. In line with fMRI studies, the right temporo-parieto-frontal network revealed activation in response to low-frequency words, and non-linguistic information played an important role in the development of language competencies in native and non-native languages. This kind of large-scale brain imaging study is feasible (because of lower cost and portability) with the help of fNIRS rather than several other imaging techniques. This finding is further corroborated by greater activation in the right superior temporal sulcus in bilingual as compared to monolingual children at the age of 5 years. Groba et al. (2018) observed that in the absence of a behavioral difference, fNIRS results show that Spanish-German bilingual children rely more on the right superior temporal sulcus than German monolingual children during adjective learning.

There are similarities between the mathematics and language studies. More difficult stimuli (here low-frequency words), different age groups and expertise groups (here bilingual children) lead to more frontal activation that supposedly underlies more domain-general processes, as in mathematics studies. FNIRS seems to be a suitable technique not only to investigate language acquisition and comprehension, but also reading and speaking both in native and foreign languages and in bilingual settings in children of different age groups.

## Future research

In this review, we present research using fNIRS in two developmentally and educationally important domains: mathematics and language. We acknowledge the importance of cognitive variables like working memory, attention, executive functions, and sensorimotoric development as well as emotional-motivational variables like anxiety, depression, motivation, and so on.

While some studies in schoolchildren have investigated basic arithmetic skills and differences between calculating and reading arithmetic problems, others have used the advantages of fNIRS, by letting children solve geometric tasks like tangram puzzles. The studies converge on their observation of a frontal-parietal network, with more frontal and/or more pronounced activation for younger children, children with less expertise or in solving more difficult arithmetic problems. With respect to language, many aspects of segmental, and suprasegmental information processing in developing children have been investigated. However, some domains are not sufficiently studied in preschoolers and schoolchildren, such as reading and speech production under different circumstances, probably because some of these paradigms are hard to realize in fMRI in children. This is one reason why, in our opinion, fNIRS should be more frequently used for studying mathematics and language.

To date, fNIRS has already contributed to investigating brain activation changes underlying several cognitive processes, particularly in infants and adults, but it has not been used as often in schoolchildren. We believe that fNIRS has the potential to become a viable and widely-used technique in educational neuroscience as well, where the target sample is children under direct academic training. It measures the hemodynamic responses and offers a procedure of high ecological validity, which makes it possible to bring fNIRS to schools so that students can be tested in a familiar environment. Therefore, its application can be extended to:

- study different cognitive functions effectively due to a silent, noiseless procedure that does not interfere with task solving and does not lead to such problems as anxiety about the unfamiliar situation or the restrictions needed in certain techniques like fMRI (Soltanlou et al., 2017a);
- investigate embodied cognition, as it is less sensitive to movements than fMRI, and enables sitting or standing positions (Bahnmueller et al., 2014). Indeed, fNIRS allows the study of cognitive development during movement. Movements can be of different types, such as finger counting and grasping, moving a dominant hand in a written production task (Artemenko et al., 2018a), or even whole body movement as in the investigation of embodied numerosity (Dackermann et al., 2017; for whole-body embodied learning);
- measure larger samples of participants for short periods of time because of lower cost (e.g., it is a one-time purchase, whereas fMRI requires additional funds per use) and portability (Dresler et al., 2009; Obersteiner et al., 2010; Sugiura et al., 2011);
- take repeated or continuous measurements for monitoring purposes (Soltanlou et al., 2018);
- combine effortlessly with other neuroimaging techniques such as EEG without any measurement interference, in order to provide a better understanding of brain mechanisms (Telkemeyer et al., 2009; Soltanlou et al., 2017a);
- investigate brain activation changes in populations with atypical development, such as in children with dyslexia or attention deficit and hyperactivity disorder (ADHD) (Moser et al., 2009; Cutini et al., 2016);
- use the method as a neurofeedback and interventional tool in cognitive development studies (Hosseini et al., 2016).

## Conclusion

Rapid constant technical improvement of fNIRS might bring us closer to bridging the gap between education and neuroscience (Ansari and Coch, 2006). FNIRS can provide novel insight into the neural basis of numerical cognition and of language acquisition or production by studying these processes in natural academic settings (Soltanlou et al., 2017a,b), where most other neuroimaging techniques, such as fMRI, are unsuitable. Furthermore, fNIRS can trace changes on the neural level during development (Artemenko et al., 2018b) and eventually the life span (Wilcox and Biondi, 2015; Gallagher et al., 2016) to understand how particular brain structures stay constant or change with maturation, experience, and learning (Gervain et al., 2011). FNIRS may also be applicable to studying the learning of mathematics and language in real life (Soltanlou et al., 2018). Usually, fNIRS is used to observe brain activation in response to cognitive and motor tasks; however, few fNIRS studies have attempted to find out how these skills are learned (Gervain et al., 2008). Furthermore, while most of the neuroimaging techniques are prone to motion artifacts, fNIRS is more flexible to movement (Bahnmueller et al., 2014; Herold et al., 2017). Notably, movement and embodied cognition can be an important intervention technique in supporting mathematics learning (e.g., physically moving the body along a number line; Dackermann et al., 2017).

Application of fNIRS has been also extended to study atypical development. Recently, Cutini et al. (2016) successfully used fNIRS for the first time to investigate hemispheric asymmetry effects of prosodic processing in children with developmental dyslexia. Furthermore, fNIRS is fortunately resilient to subtle movements, which makes it suitable for measuring children with ADHD (Weber et al., 2005; Moser et al., 2009). Moreover, fNIRS was shown to be an effective tool for neurofeedback in ADHD children (Marx et al., 2014; Blume et al., 2017) and in improving cognitive and motor performance (Hosseini et al., 2016; Lapborisuth et al., 2017). These studies suggest a good outlook for the application of fNIRS as a neurofeedback tool in cognitive development training, including mathematics and language in children with developmental dyscalculia and dyslexia. In general, because of the very fast development of fNIRS devices, analysis toolboxes, and its reliable findings in different fields, the fNIRS technique can be regarded as a versatile and promising instrument in educational neuroscience.

## Author contributions

MS, MAS, and TD: wrote the first draft of the manuscript; all authors edited the manuscript in various versions and commented on it.

### Conflict of interest statement

The authors declare that the research was conducted in the absence of any commercial or financial relationships that could be construed as a potential conflict of interest.
